# Supplementary material for: Programming gene expression in multicellular organisms for physiology modulation through engineered bacteria
Source: Nat Commun. 2021 May 11;12:2689. doi: 10.1038/s41467-021-22894-7 (PMC8113242; doi:10.1038/s41467-021-22894-7)
Supplement: Supplementary file 1 — Supplementary Information [file 41467_2021_22894_MOESM1_ESM.pdf]

Supplementary Information

Programming gene expression in multicellular organisms for physiology modulation through engineered bacteria

Authors: Baizhen Gao[1] and Qing Sun[1,*]

Affiliations:

[1] Department of Chemical Engineering, Texas A&M University, College Station, TX 77840

*Corresponding author. Email: sunqing@tamu.edu

This PDF file includes:

Materials and Methods

Supplementary Text

Figs. S1 to S6

Tables S1

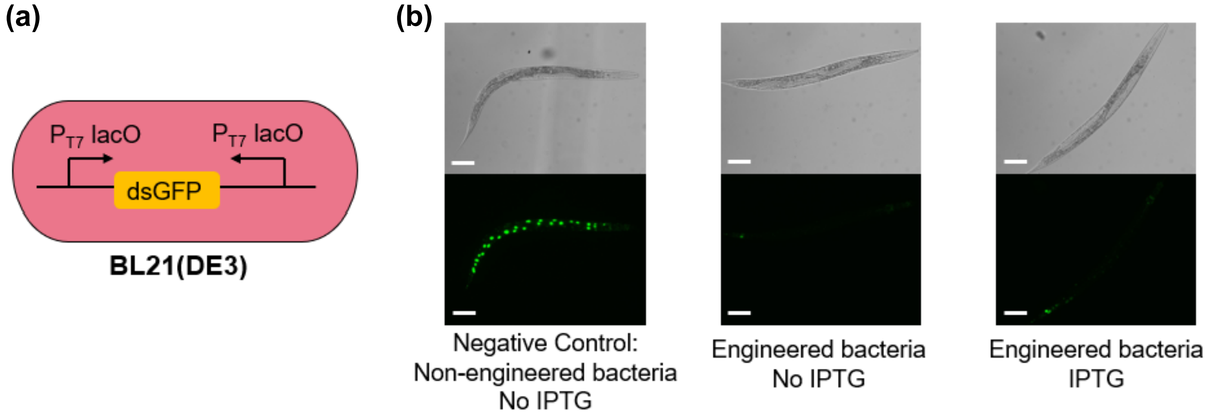

Supplementary Figure 1. *C. elegans* GFP expression with and without *E. coli* harboring dsRNA for GFP. (**a**) Schematic diagram of *E. coli* BL21(DE3) producing double-stranded GFP RNA. (**b**) *C. elegans* fed with: (left) *E. coli* BL21(DE3) without plasmid; (middle) *E. coli* BL21(DE3) containing the plasmid producing dsRNA for GFP but not induced by IPTG; and (right) *E. coli* BL21(DE3) containing the plasmid and induced by 1 mM IPTG. A 750 bp fragment for *gfp* from *C. elegans* SD1084 was inserted between two T7/LacO that are in reciprocal orientation on a pET24a plasmid vector. The plasmid was then transformed into *E. coli* BL21(DE3) and fed to SD1084 worms cultured on NGM plates with or without 1mM IPTG. The *E. coli* BL21(DE3) without any plasmids was also fed to SD1084 worms as a negative control. Scale bar, 100 µm. The experiment was repeated 3 times independently for a total of 15 worms with similar results.

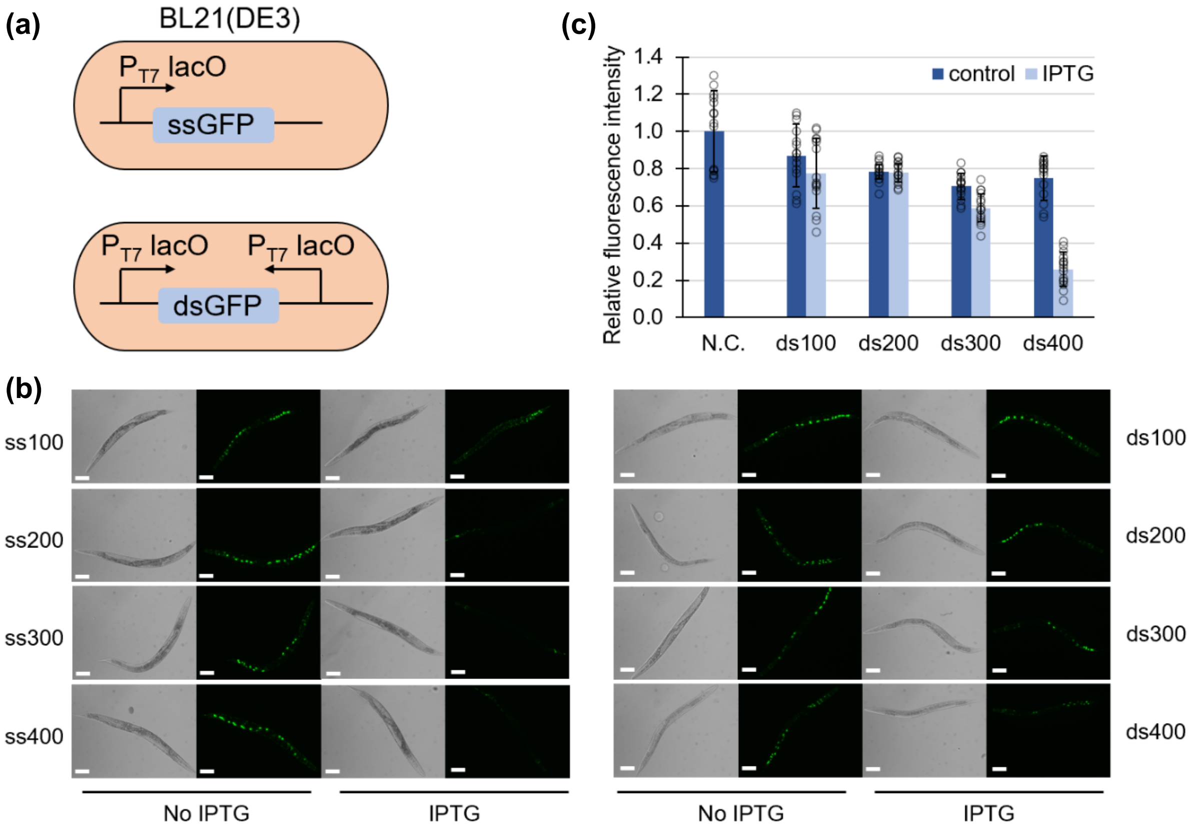

Supplementary Figure 2. Microcopy images and fluorescence intensity quantification of *C. elegans* GFP expression. (**a**) Schematic of *E. coli* producing single-stranded or double-stranded *gfp* RNA in response to IPTG. (**b**) Optical and fluorescent images of *C. elegans* SD1084 fed with engineered *E. coli* producing *gfp* RNA with lengths ranging from 100bp to 400bp with or without 1mM IPTG induction. Scale bar, 100 μm. The experiment was repeated 3 times independently for a total of 15 worms with similar results. (**c**) Fluorescence intensity quantification of *C. elegans* SD1084 fed with 100bp to 400bp *gfp* RNA in double-stranded format. (Error bars represent the mean ± standard deviation for n=15 worms over three independent experiments)

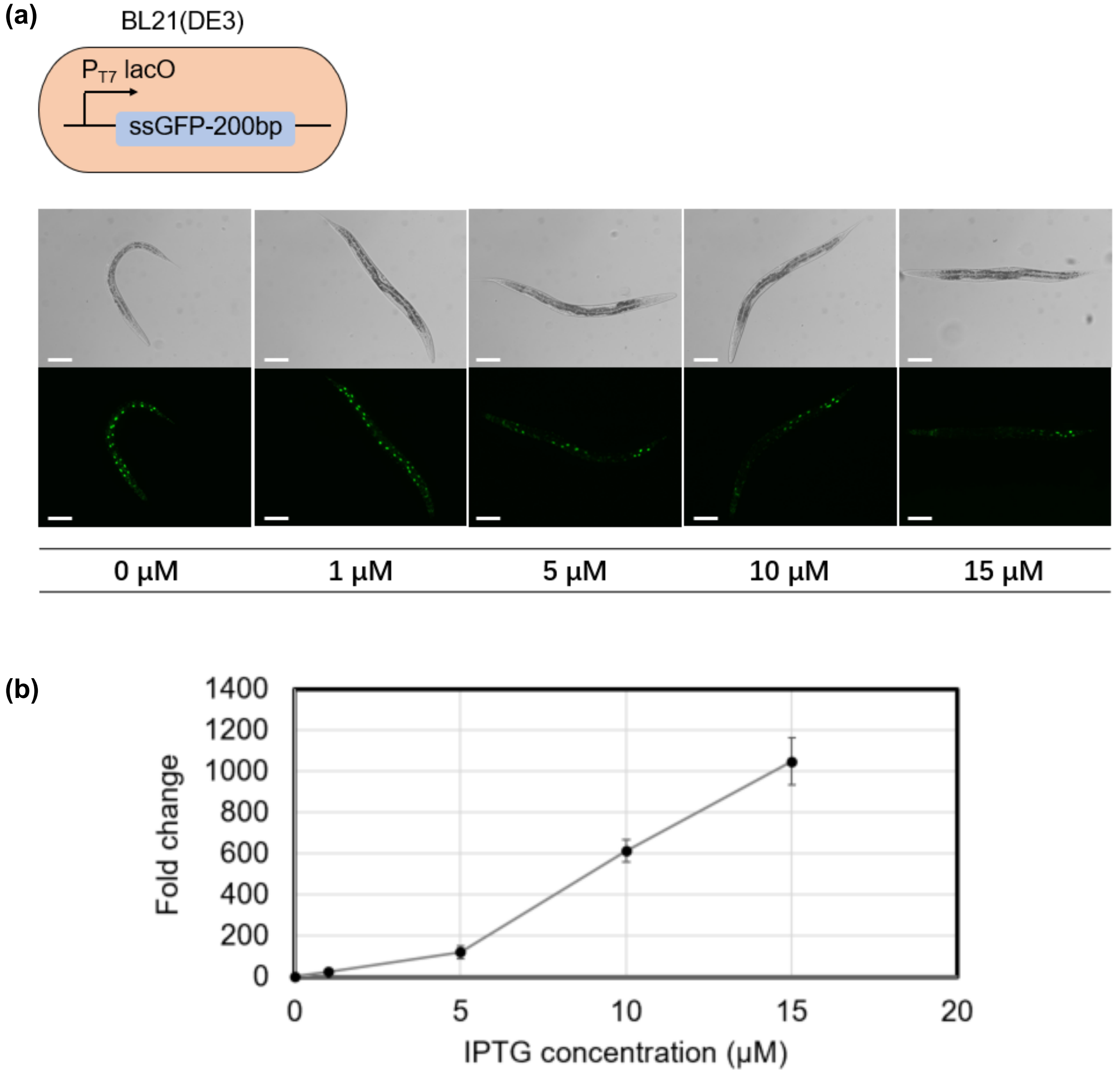

Supplementary Figure 3. Images of *C. elegans gfp* expression in response to IPTG, and RNA synthesis quantification results by RT-qPCR. (**a**) Schematic of *E. coli* producing 200bp single-stranded *gfp* RNA in response to IPTG, and optical and fluorescent images of *C. elegans* SD1084 fed with engineered *E. coli* producing 200bp single-stranded *gfp* RNA in response to IPTG concentrations ranging from 0 to 15 µM. Scale bar, 100 µm. The experiment was repeated 3 times independently for a total of 15 worms with similar results. (**b**) RT-qPCR results showed the fold change of the bacterial 200bp single-stranded *gfp* RNA synthesis at indicated IPTG concentrations relative to the control group with no IPTG. (Error bars represent the mean ± standard deviation for n=9 replicates from 3 biologically independent samples)

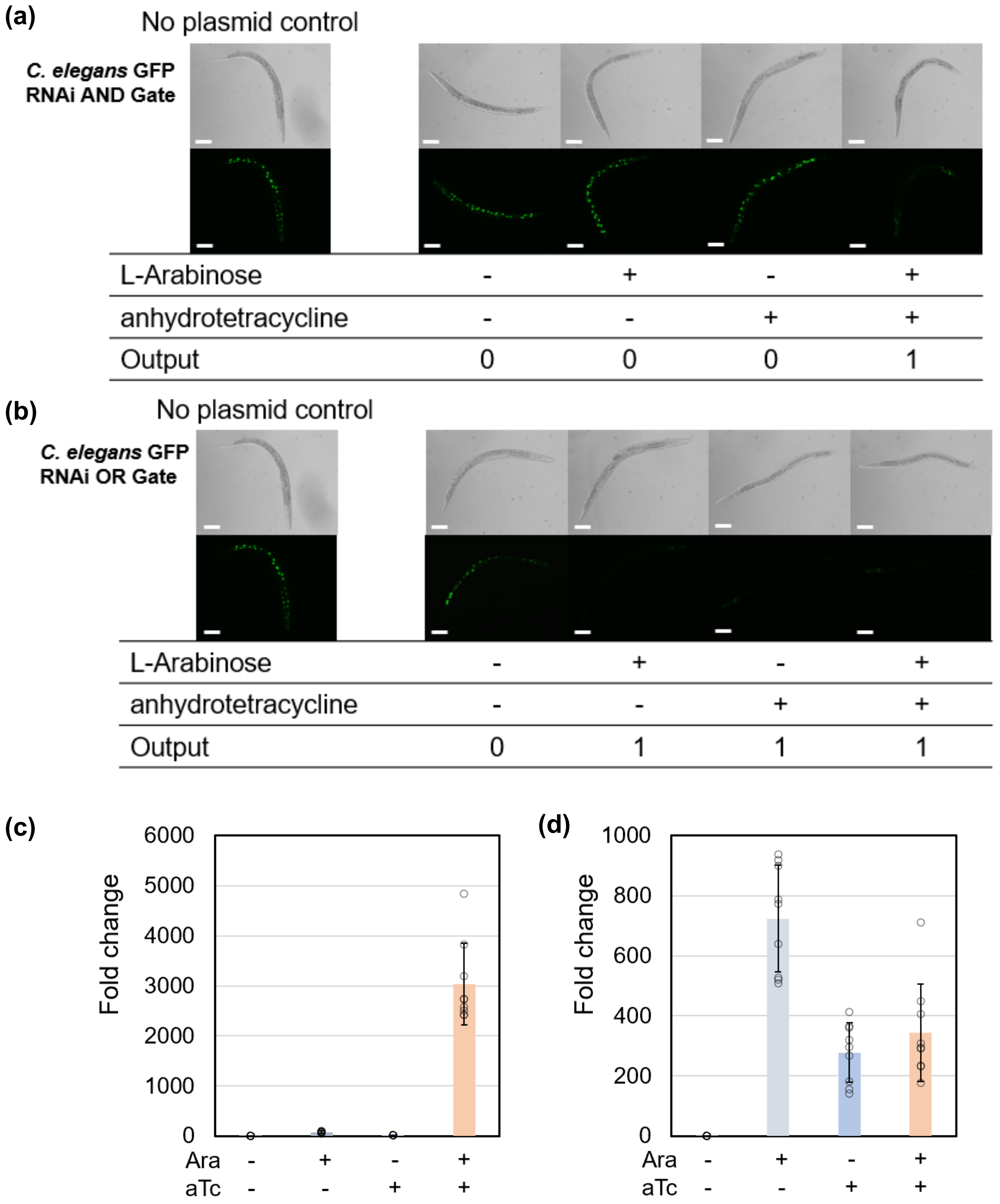

Supplementary Figure 4. Optical and fluorescence microcopy images of *C. elegans* GFP expression. (**a**) AND-gate and (**b**) OR-gate controlled *C. elegans* GFP expression in response to no inducers, 0.2 mg/mL of L-arabinose, 0.1 µg/mL of anhydrotetracycline, and both inducers. Scale bar, 100 µm. The experiment was repeated 3 times independently for a total of 15 worms with similar results. (**c**) AND-gate and (**d**) OR-gate controlled 200bp single-stranded *gfp* RNA synthesis levels relative to the control group with no IPTG. RT-qPCR was used to show the *gfp* RNA levels. (Error bars represent the mean ± standard deviation for n=9 replicates from 3 biologically independent samples)

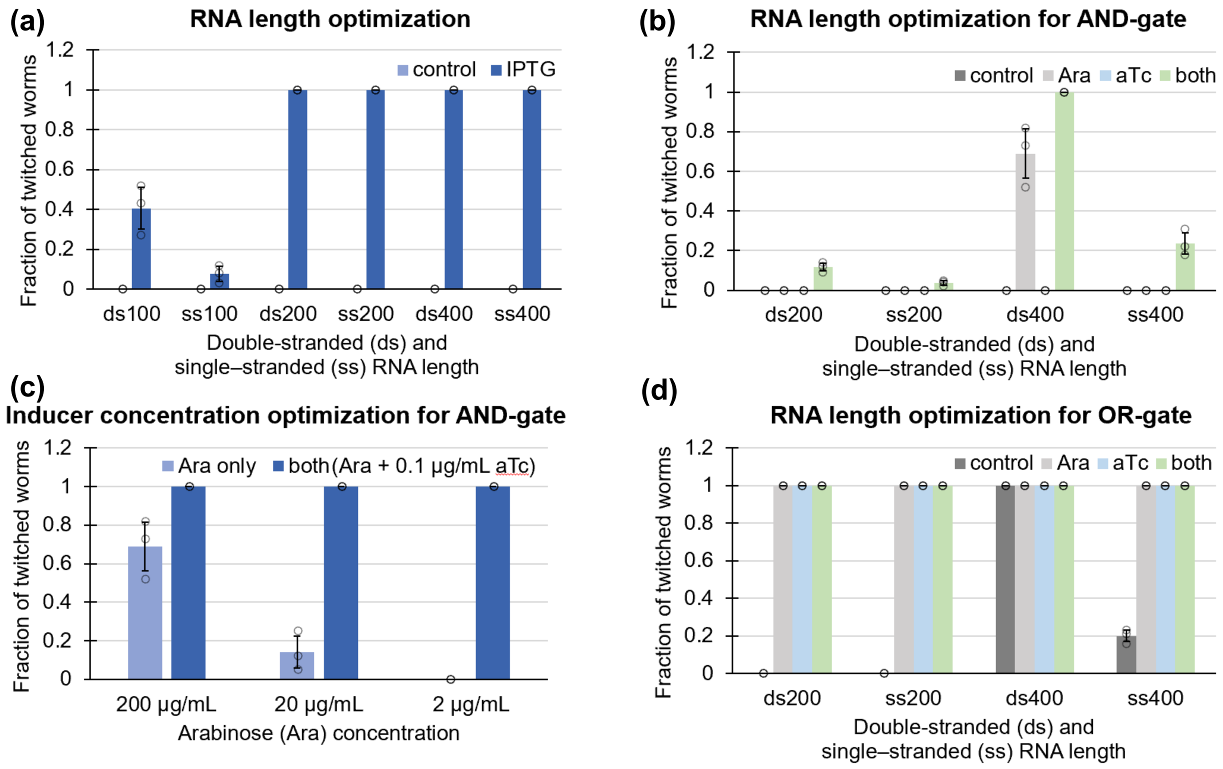

Supplementary Figure 5. Optimization of RNA lengths and inducer concentrations. **(a)** Optimization of *unc-22* RNA length and format for inducing *C. elegans* twitching phenotype by 1 mM IPTG. The fraction of twitched worms were counted for worms fed on BL21(DE3) synthesizing double or single-stranded *unc-22* RNA with lengths 100, 200, and 400 bp. Both 200 bp and 400 bp *unc-22* dsRNA and ssRNA achieved "ON" and "OFF" modulation of worm twitching phenotype (**b**) Length optimization of *unc-22* RNA for *C. elegans* twitching phenotype controlled by AND gate. BL21 co-transformed with the AND-gate circuit plasmid and the indicated RNA synthesizing plasmid were fed to N2 worms with no inducer (control), 0.2 mg/mL L-arabinose (Ara), 0.1 µg/mL anhydrotetracycline (aTc), or both inducers. Bacteria producing ds400 led to complete twitching phenotype in *C. elegans* with both inducers, but also caused twitching phenotype in around 70% of the worms with 0.2 mg/mL Ara only sample. Therefore, the Ara concentration was further optimized with ds400 RNA and 0.1 µg/mL aTc in C. (**c**) Inducer concentration optimization for *C. elegans* twitching phenotype controlled by AND gate. A series of Ara concentration was used (indicated on the x-axis) in combination with 0.1 µg/mL aTc. As a result, 2 µg/mL Ara and 0.1 µg/mL aTc together were able to achieve full "ON/OFF" status with AND-gate and ds400 *unc-22* RNA for *C. elegans* twitching phenotype control. (**d**) Length optimization for OR gate on *C. elegans* twitching. BL21 co-transformed with the OR-gate circuit plasmid and RNA synthesizing plasmid were fed to N2 worms with no inducer (control), 0.2 mg/mL Ara, 0.1 µg/mL aTc, or both inducers. Since our initial try with both ds400 and ss400 showed the OR-gate profile using 0.2 mg/mL Ara and 0.1 µg/mL aTc, the inducer concentrations were not further optimized. (Error bars represent the mean ± standard deviation for n=200 worms over three independent experiments)

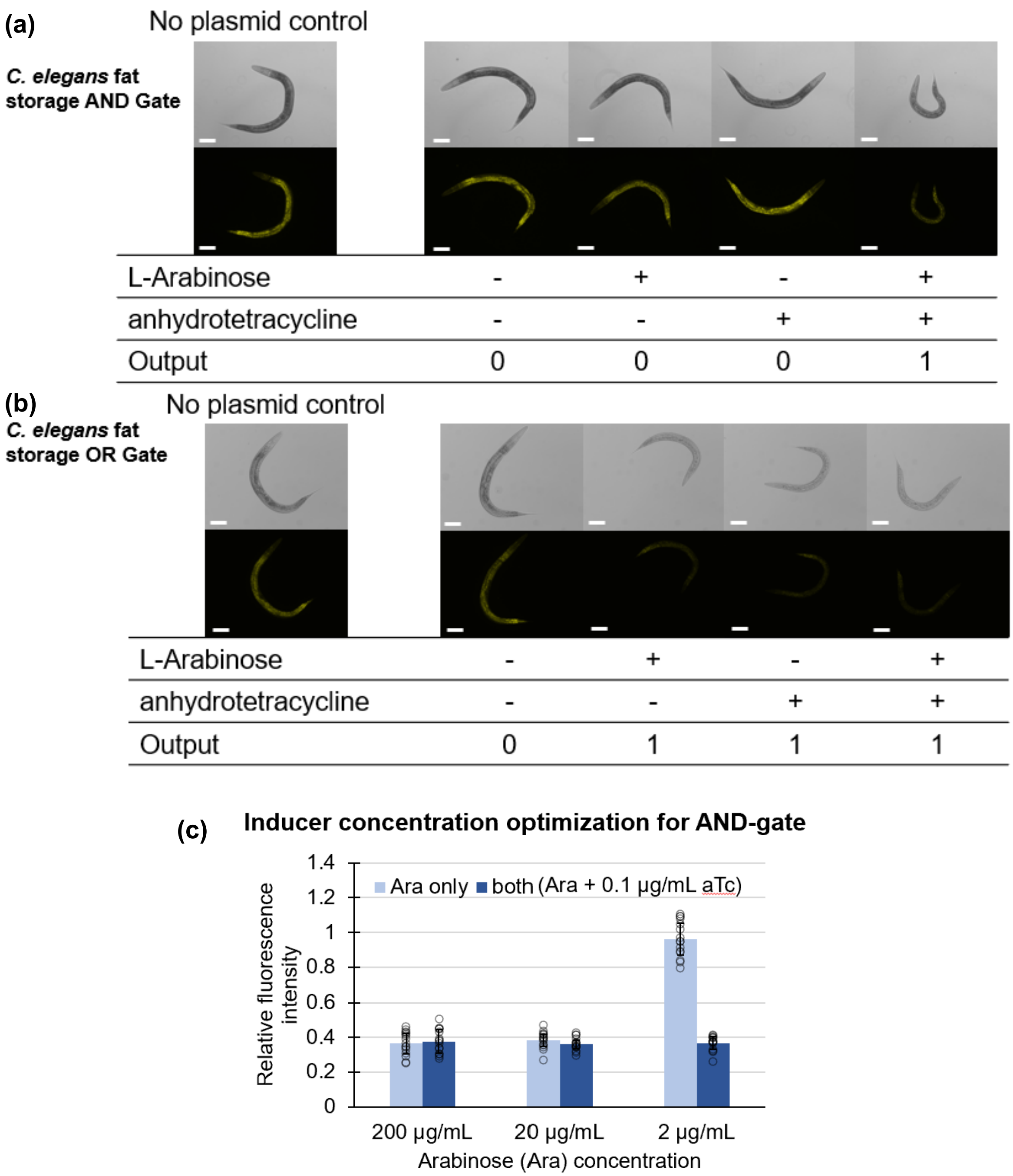

Supplementary Figure 6. Optical and fluorescence microscopy images of *C. elegans* fat storage. (**a**) AND-gate controlled *C. elegans* fat storage in response 2 µg/mL Ara, 0.1 µg/mL of aTc, or both inducers. (**b**) OR-gate controlled *C. elegans* fat storage indicated by Nile Red staining in response to 0.2 mg/mL of L-arabinose (Ara), 0.1 µg/mL of anhydrotetracycline (aTc), or both inducers. Since our initial try with full length sbp-1, 0.2 mg/mL Ara and 0.1 µg/mL aTc gave an OR-gate profile, the inducer concentrations and RNA length were not further optimized. Scale bar, 100 µm. The experiment was repeated 3 times independently for a total of 15 worms with similar results. (**c**) Inducer concentration optimization for *C. elegans* fat-storage programmed by AND

gate. The initial AND-gate controlled fat-storage with 0.2 mg/mL Ara and 0.1 µg/mL aTc didn't work because 0.2 mg/mL of Ara alone led to a similar level of silencing effect as with both inducers. To optimize the inducer concentration, Ara concentration was diluted to 20 µg/mL and 2 µg/mL while aTc concentration was kept constant at 0.1 µg/mL. In the end, 2 µg/mL Ara and 0.1 µg/mL aTc were picked for the AND-gate experiments. Since full length sbp-1 worked after inducer concentration optimization, RNA length experiments were not further conducted. (Error bars represent the mean ± standard deviation for n=15 worms over three independent experiments)

Supplementary Table 1. Sequences of DNA used in this paper

Name	Type	Sequence
GFP-100bp	gene	gatatcggagaagaacttttcactggagttgtcccaattcttgttgaattagatggtgatgttaa tgggcacaaattttctgtcagtggagagggtgaag
GFP-200bp	gene	gatatcggagaagaacttttcactggagttgtcccaattcttgttgaattagatggtgatgttaa tgggcacaaattttctgtcagtggagagggtgaaggtgatgcaacatacggaaaacttaccc ttaaatttatttgcactactggaaaactacctgttccatgggtaagtttaaacatatatatactaac taaccc
GFP-300bp	gene	gatatcggagaagaacttttcactggagttgtcccaattcttgttgaattagatggtgatgttaa tgggcacaaattttctgtcagtggagagggtgaaggtgatgcaacatacggaaaacttaccc ttaaatttatttgcactactggaaaactacctgttccatgggtaagtttaaacatatatatactaac taaccctgattatttaaattttcagccaacacttgtcactactttctgttatggtgttcaatgcttctc gagatacccagatcatatgaaacggcatgactttttc
GFP-400bp	gene	gatatcggagaagaacttttcactggagttgtcccaattcttgttgaattagatggtgatgttaa tgggcacaaattttctgtcagtggagagggtgaaggtgatgcaacatacggaaaacttaccc ttaaatttatttgcactactggaaaactacctgttccatgggtaagtttaaacatatatatactaac taaccctgattatttaaattttcagccaacacttgtcactactttctgttatggtgttcaatgcttctc gagatacccagatcatatgaaacggcatgactttttcaagagtgccatgcccgaaggttatgt acaggaaagaactatatttttcaaagatgacgggaactacaagacacgtaagtttaaacagtt cggtactaact
unc-22-200bp	gene	agcttctcaacgatgtagtttgtgattggcgatcctccatcagatgatggtggcatccagtaaa gagtgcagcagtctggtgcaatatcctctactcggatgtgttgtggtgaagctggtttatcaac aaccttcaatctgatactagcagaatcacttccttgactattttcaagttcaacaaagtagagtcc accatc
unc-22-400bp	gene	agcttctcaacgatgtagtttgtgattggcgatcctccatcagatgatggtggcatccagtaaa gagtgcagcagtctggtgcaatatcctctactcggatgtgttgtggtgaagctggtttatcaac aaccttcaatctgatactagcagaatcacttccttgactattttcaagttcaacaaagtagagtcc accatcagtaagttcagacttctcgatcgtcaatgtagccaagaagtcactgtactcaaccttca ctcttggatcagttgggctgatcttattctcacccttcgagaaagtgactttcggagcaggtga agcagcaaatggaacaagaatctttgcacgttctccagcgtaagcgaggatatcgcgagta agcattcccatgttga
sbp-1	gene	aattggatgatattgcgcgtgagtttttccctatatgcctacctcatgcctacctgttcgcctaca tgcctacgtacctgcctaccttgtccctatctgcgtacgcttacctgcctacctcatacctcctggt ttgcgcctacacaccaggcttacaccaaaaacaagcattcaatcaaaacacaaaaaataatttt caaaaacccaaaacacctacctcatgcctaccagcctagcccatgcctacgtgcctacctcatg cctacctgcctagcccttgcctacctgccaacctcatgcctacctgctcaacacaacttacctac gcccataaacttgctcattgaaggaatataatttctatagtatattagtttagcagtaggcaggc aggcttgcattttcggtgcctatagttctaattttcagcatttccaaataacgacccgctcgatttt gacatggagcacaactggcaagagcccggaccatcacaactaccggatccatcaattcccg

		gaaatcaacacagtccgccacaggaatattatgatattgatgggtgagtttggctagtgtact<br>gtgaggtactatttcgcataggcctgaaaaattatgcctgcctacaaggccacctgtgcctgc<br>ctacgtgccaactactgacgtaaagcttaggctactaaaacaataggtatattaaaaaatataa<br>gtaatgtaggtaggcaagcctaccttccgtggcatcttttcatttgtcaggagagatggcagg<br>cgtaggc
T7 RNAP N-terminus	gene	atgaacacgattaacatcgctaagaacgacttctctgacatcgaactggctgctatcccgttca<br>acactctggctgaccattacggtgagcgtttagctcgcgaacagttggcccttgagcatgagt<br>cttacgagatgggtgaagcacgcttccgcaagatgtttgagcgtcaacttaaagctggtgag<br>gttgcggataacgctgccgccaagcctctcatcactaccctactccctaagatgattgcacgca<br>tcaacgactggtttgaggaagtgaaagctaagcgcggcaagcgcccgacagccttccagtt<br>cctgcaagaaatcaagccggaagccgtagcgtacatcaccattaagaccactctggcttgcc<br>taaccagtgctgacaatacaaccgttcaggctgtagcaagcgcaatcggtcgggccattga<br>ggacgaggctcgcttcggtcgtatccgtgaccttgaagctaagcacttcaagaaaaacgttg<br>aggaacaactcaacaagcgcgtagggcacgtctacaagtaa
T7 RNAP C-terminus	gene	atgaaagcatttatgcaagttgtcgaggctgacatgctctctaagggtctactcggtggcgag<br>gcgtggtcttcgtggcataaggaagactctattcatgtaggagtacgctgcatcgagatgctc<br>attgagtcaaccggaatggttagcttacaccgccaaaatgctggcgtagtaggtcaagactct<br>gagactatcgaactcgcacctgaatacgctgaggctatcgcaacccgtgcaggtgcgctgg<br>ctggcatctctccgatgttccaaccttgcgtagttcctcctaagccgtggactggcattactggt<br>ggtggctattgggctaacggtcgtcgtcctctggcgctggtgcgtactcacagtaagaaagc<br>actgatgcgctacgaagacgtttacatgcctgaggtgtacaaagcgattaacattgcgcaaa<br>acaccgcatggaaaatcaacaagaaagtcctagcggtcgccaacgtaatcaccaagtggaa<br>gcattgtccggtcgaggacatccctgcgattgagcgtgaagaactcccgatgaaaccggaa<br>gacatcgacatgaatcctgaggctctcaccgcgtggaaacgtgctgccgctgctgtgtaccg<br>caaggacaaggctcgcaagtctcgccgtatcagccttgagttcatgcttgagcaagccaata<br>agtttgctaaccataaggccatctggttcccttacaacatggactggcgcggtcgtgtttacgc<br>tgtgtcaatgttcaacccgcaaggtaacgatatgaccaaaggactgcttacgctggcgaaag<br>gtaaaccaatcggtaaggaaggttactactggctgaaaatccacggtgcaaactgtgcggg<br>tgtcgataaggttccgttccctgagcgcatcaagttcattgaggaaaaccacgagaacatcat<br>ggcttgcgctaagtctccactggagaacacttggtgggctgagcaagattctccgttctgcttc<br>cttgcgttctgctttgagtacgctggggtacagcaccacggcctgagctataactgctcccttc<br>cgctggcgtttgacgggtcttgctctggcatccagcacttctccgcgatgctccgagatgagg<br>taggtggtcgcgcggttaacttgcttcctagtgaaaccgttcaggacatctacgggattgttgc<br>taagaaagtcaacgagattctacaagcagacgcaatcaatgggaccgataacgaagtagtt<br>accgtgaccgatgagaacactggtgaaatctctgagaaagtcaagctgggcactaaggcac<br>tggctggtcaatggctggcttacggtgttactcgcagtgtgactaagcgttcagtcatgacgc<br>tggcttacgggtccaaagagttcggcttccgtcaacaagtgctggaagataccattcagcca<br>gctattgattccggcaagggtctgatgttcactcagccgaatcaggctgctggatacatggct<br>aagctgatttgggaatctgtgagcgtgacggtggtagctgcggttgaagcaatgaactggc<br>ttaagtctgctgctaagctgctggctgctgaggtcaaagataagaagactggagagattcttc<br>gcaagcgttgcgctgtgcattgggtaactcctgatggtttccctgtgtggcaggaatacaaga<br>agcctattcagacgcgcttgaacctgatgttcctcggtcagttccgcttacagcctaccattaac<br>accaacaaagatagcgagattgatgcacacaaacaggagtctggtatcgctcctaactttgta

		cacagccaagacggtagccaccttcgtaagactgtagtgtgggcacacgagaagtacgga<br>atcgaatcttttgcactgattcacgactccttcggtaccattccggctgacgctgcgaacctgtt<br>caaagcagtgcgcgaaactatggttgacacatatgagtcttgtgatgtactggctgatttctac<br>gaccagttcgctgaccagttgcacgagtctcaattggacaaaatgccagcacttccggctaa<br>aggtaacttgaacctccgtgacatcttagagtcggacttcgcgttcgcgtaa
araC	gene	atggctgaagcgcaaaatgatcccctgctgccgggatactcgtttaatgcccatctggtggcg<br>ggtttaacgccgattgaggccaacggttatctcgatttttttatcgaccgaccgctgggaatga<br>aaggttatattctcaatctcaccattcgcggtcaggggggtggtgaaaaatcagggacgagaa<br>tttgtttgccgaccgggtgatattttgctgttcccgccaggagagattcatcactacggtcgtca<br>tccggaggctcgcgaatggtatcaccagtgggcttactttcgtccgcgcgcctactggcatga<br>atggcttaactggccgtcaatatttgccaatacggggttctttcgcccggatgaagcgcacca<br>gccgcatttcagcgacctgtttgggcaaatcattaacgccgggcaaggggaagggcgctat<br>tcggagctgctggcgataaatctgcttgagcaattgttactgcggcgcatggaagcgattaa<br>cgagtcgctccatccaccgatggataatcgggtacgcgaggcttgtcagtacatcagcgatc<br>acctggcagacagcaattttgatatcgccagcgtcgcacagcatgtttgcttgtcgccgtcgc<br>gtctgtcacatcttttccgccagcagttagggattagcgtcttaagctggcgcgaggaccaac<br>gtatcagccaggcgaagctgcttttgagcaccacccggatgcctatcgccaccgtcggtcgc<br>aatgttggttttgacgatcaactctatttctcgcgggtatttaaaaaatgcaccggggccagcc<br>cgagcgagttccgtgccggttgtgaagaaaaagtgaatgatgtagccgtcaagttgtcataa
TetR	gene	atgtctagattagataaaagtaaagtgattaacagcgcattagagctgcttaatgaggtcgga<br>atcgaaggtttaacaacccgtaaactcgcccagaagctaggtgtagagcagcctacattgtat<br>tggcatgtaaaaaataagcgggctttgctcgacgccttagccattgagatgttagataggcac<br>catactcacttttgccctttagaaggggaaagctggcaagattttttacgtaataacgctaaaa<br>gttttagatgtgctttactaagtcatcgcgatggagcaaaagtacatttaggtacacggcctac<br>agaaaaacagtatgaaactctcgaaaatcaattagcctttttatgccaacaaggtttttcactag<br>agaatgcattatatgcactcagcgctgtggggcattttactttaggttgcgtattggaagatca<br>agagcatcaagtcgctaaagaagaaagggaaacacctactactgatagtatgccgccattat<br>tacgacaagctatcgaattatttgatcaccaaggtgcagagccagccttcttattcggccttga<br>attgatcatatgcggattagaaaaacaacttaaatgtgaaagtgggtcttaa
T7-<br>Lysozyme	gene	atggctcgtgtacagtttaaacaacgtgaatctactgacgcaatctttgttcactgctcggctac<br>caagccaagtcagaatgttggtgtccgtgagattcgccagtggcacaaagagcagggttg<br>gctcgatgtgggataccactttatcatcaagcgagacggtactgtggaggcaggacgagat<br>gagatggctgtaggctctcacgctaagggttacaaccacaactctatcggcgtctgccttgtt<br>ggtggtatcgacgataaaggtaagttcgacgctaactttacgccagcccaaatgcaatccctt<br>cgctcactgcttgtcacactgctggctaagtacgaaggcgctggtcttcgcgcccatcatgag<br>gtggcgccgaaggcttgcccttcgttcgaccttaagcgttggtgggagaagaacgaactgg<br>tcacttctgaccgtggataa

LacI	gene	gtgaaaccagtaacgttatacgatgtcgcagagtatgccggtgtctcttatcagaccgtttccc gcgtggtgaaccaggccagccacgtttctgcgaaaacgcgggaaaaagtggaagcggcg atggcggagctgaattacattcccaaccgcgtggcacaacaactggcgggcaaacagtcgt tgctgattggcgttgccacctccagtctggccctgcacgcgccgtcgcaaattgtcgcggcg attaaatctcgcgccgatcaactgggtgccagcgtggtggtgtcgatggtagaacgaagcg gcgtcgaagcctgtaaagcggcggtgcacaatcttctcgcgcaacgcgtcagtgggctgat cattaactatccgctggatgaccaggatgccattgctgtggaagctgcctgcactaatgttccg gcgttatttcttgatgtctctgaccagacacccatcaacagtattattttctcccatgaagacggt acgcgactgggcgtggagcatctggtcgcattgggtcaccagcaaatcgcgctgttagcgg gcccattaagttctgtctcggcgcgtctgcgtctggctggctggcataaatatctcactcgcaa tcaaattcagccgatagcggaacgggaaggcgactggagtgccatgtccggttttcaacaa accatgcaaatgctgaatgagggcatcgttcccactgcgatgctggttgccaacgatcagat ggcgctgggcgcaatgcgcgccattaccgagtccgggctgcgcgttggtgcggatatctcg gtagtgggatacgacgataccgaagacagctcatgttatatcccgccgttaaccaccatcaaa caggattttcgcctgctggggcaaaccagcgtggaccgcttgctgcaactctctcagggcca ggcggtgaagggcaatcagctgttgcccgtctcactggtgaaaagaaaaaccaccctggc gcccaatacgcaaaccgcctctccccgcgcgttggccgattcattaatgcagctggcacgac aggtttcccgactggaaagcgggcagtga
pTet	promoter	gttgacactctatcgttgatagagttattttaccactccctatcagtgatagagaa
pBAD	promoter	aagaaaccaattgtccatattgcatcagacattgccgtcactgcgtcttttactggctcttctcgc taaccaaaccggtaaccccgcttattaaaagcattctgtaacaaagcgggaccaaagccatg acaaaaacgcgtaacaaaagtgtctataatcacggcagaaaagtccacattgattatttgcac ggcgtcactctttgctatgccatagcatttttatccataagattagcggatcctacctgacgctttt tatcgcaactctctactgtttctccat
proD	promoter	cacagctaacaccacgtcgtccctatctgctgccctaggtctatgagtggttgctggataacttt acgggcatgcataaggctcgtataatatattcagggagaccacaacggtttccctctacaaat aattttgtttaacttt
Forward pET24a	primer	caccgcctacatacctcgctctgc
pET24a reverse	primer	gcagagcgaggtatgtaggcggtg
QSOligo13-GFP forward pet	primer	gatatcggagaagaacttttcactgga
QSOligo14-pET24a reverse-dsGFP	primer	cagtgaaaagttcttctccgatatctctagaggggaattgttatccgctcacaattc
QSOligo16-pET24a forward ds template PCR2	primer	tatccgctcacaattcccctatagtgagtcgtattatgagatccggctgctaacaaagcc

QSOligo42-sbp-1 forward	primer	catgaattcattcgagggagacgtccc
QSOligo99-sbp-1 reverse	primer	catgaattcctgatgtggagtcatcgc
QSOligo117-GFP100bpR	primer	aggggaattgtgagcggataacaattcccttcaccctctccactgacagaaaa
QSOligo118-GFP200bpR	primer	gaattgtgagcggataacaattccgggttagttagtatatatatgtttaaacttacccat
QSOligo119-GFP300bpR	primer	gaattgtgagcggataacaattccgaaaaagtcatgccgtttcatatgatctg
QSOligo120-GFP400bpR	primer	gaattgtgagcggataacaattccagttagtaccgaactgtttaaacttacgtgt
QSOligo123-ssGFP400bpR	primer	cctttcgggctttgttagcagccggatctcaagttagtaccgaactgtttaaacttacgt
QSOligo124-ssGFP300bpR	primer	cctttcgggctttgttagcagccggatctcagaaaaagtcatgccgtttcatatgatctg
QSOligo125-ssGFP200bpR	primer	cgggctttgttagcagccggatctcagggttagttagtatatatatgtttaaacttaccc
QSOligo126-ssGFP100bpR	primer	cctttcgggctttgttagcagccggatctcacttcaccctctccactgacagaaa
QSOligo130-ProD reverse-TetR	primer	cttttatctaatctagacataaagttaaacaaaattatttgtagagggaaaccgttgtgg
QSOligo131-TetR Forward	primer	atgtctagattagataaaagtaaagtgattaacagcgcat
QSOligo132-TetP-R	primer	cattaattcctaattgctagcattgtacctaggactgag

QSOligo133 -ProD_for-TetR	primer	ctagcaattaggaattaatgcacagctaacaccacgtcgtccct
QSOligo134 -ProD-Reverse-AraC	primer	tcattttgcgcttcagccataaagttaaacaaaattatttgtagagggaaaccgttgtgg
QSOligo135 -araC-Forward	primer	atggctgaagcgcaaaatgatcccc
QSOligo136 -ProD-Forward-AraP	primer	atggcgggagtatgaaaagtcacagctaacaccacgtcgtccct
QSOligo137 -Arareverse	primer	acttttcatactcccgccattcagagaagaaacc
BGOligo36-unc22 forward	primer	agcttctcaacgatgtagtttgtgattggc
BGOligo37-LacO-unc-22	primer	aactacatcgttgagaagcttctagaggggaattgttatccgctcacaattc
BGoligo43-dsunc-22-200 LacO rev	primer	gaattgtgagcggataacaattccgatggtggactctactttgttgaacttgaaaatagt
BGOligo44-dsunc-22-400 LacO rev	primer	aggggaattgtgagcggataacaattcctcaacatgggaatgcttactcgcgatatc
BGOligo46-ssunc-22-200 rev	primer	cgggctttgttagcagccggatctcagatggtggactctactttgttgaacttgaaaata
BGOligo47-ssunc-22-400 rev	primer	cgggctttgttagcagccggatctcatcaacatgggaatgcttactcgcg
BGOligo49-ssunc-22-100 rev	primer	cctttcgggctttgttagcagccggatctcatccgagtagaggatattgcaccagact
BGOligo50-dsunc-22-100 rev	primer	aggggaattgtgagcggataacaattcctccgagtagaggatattgcaccagact

gfp-F	primer	CGGAGAAGAACTTTTCACTGG
gfp-R	primer	GGTAAGTTTTCCGTATGTTGC
cysG-F	primer	GATCGCGACTGTCTGATTG
cysG-R	primer	CGGTGAACTGTGGAATAAACG
