# Supplementary material for: Programming gene expression in multicellular organisms for physiology modulation through engineered bacteria
Source: Nat Commun. 2021 May 11;12:2689. doi: 10.1038/s41467-021-22894-7 (PMC8113242; doi:10.1038/s41467-021-22894-7)
Supplement: Supplementary file 2 — Reporting Summary [file 41467_2021_22894_MOESM2_ESM.pdf]

nature research

Corresponding author(s): Dr. Qing Sun

Last updated by author(s): Mar 10, 2021

Reporting Summary

Nature Research wishes to improve the reproducibility of the work that we publish. This form provides structure for consistency and transparency in reporting. For further information on Nature Research policies, see our Editorial Policies and the Editorial Policy Checklist.

Statistics

For all statistical analyses, confirm that the following items are present in the figure legend, table legend, main text, or Methods section.

n/a	Confirmed	
☐	☒	The exact sample size (n) for each experimental group/condition, given as a discrete number and unit of measurement
☐	☒	A statement on whether measurements were taken from distinct samples or whether the same sample was measured repeatedly
☐	☒	The statistical test(s) used AND whether they are one- or two-sided<br>*Only common tests should be described solely by name; describe more complex techniques in the Methods section.*
☒	☐	A description of all covariates tested
☐	☒	A description of any assumptions or corrections, such as tests of normality and adjustment for multiple comparisons
☐	☒	A full description of the statistical parameters including central tendency (e.g. means) or other basic estimates (e.g. regression coefficient) AND variation (e.g. standard deviation) or associated estimates of uncertainty (e.g. confidence intervals)
☐	☒	For null hypothesis testing, the test statistic (e.g. F, t, r) with confidence intervals, effect sizes, degrees of freedom and P value noted<br>*Give P values as exact values whenever suitable.*
☒	☐	For Bayesian analysis, information on the choice of priors and Markov chain Monte Carlo settings
☒	☐	For hierarchical and complex designs, identification of the appropriate level for tests and full reporting of outcomes
☒	☐	Estimates of effect sizes (e.g. Cohen's d, Pearson's r), indicating how they were calculated

Our web collection on statistics for biologists contains articles on many of the points above.

Software and code

Policy information about availability of computer code

Data collection	LightCycler 96 software was used for collecting qPCR data. AxioVision40 was used to acquire images taken under microscope Axiovert 200M.
Data analysis	ImageJ was used for fluorescence intensity quantification

For manuscripts utilizing custom algorithms or software that are central to the research but not yet described in published literature, software must be made available to editors and reviewers. We strongly encourage code deposition in a community repository (e.g. GitHub). See the Nature Research guidelines for submitting code & software for further information.

Data

Policy information about availability of data

All manuscripts must include a data availability statement. This statement should provide the following information, where applicable:
- Accession codes, unique identifiers, or web links for publicly available datasets
- A list of figures that have associated raw data
- A description of any restrictions on data availability

Data supporting the findings of this study are available within the paper and its Supplementary Information files. Source data are provided with this paper. The datasets generated and analyzed during the current study are available from the corresponding author upon request. A reporting summary for this article is available as a Supplementary Information file.

Field-specific reporting

Please select the one below that is the best fit for your research. If you are not sure, read the appropriate sections before making your selection.

☒ Life sciences ☐ Behavioural & social sciences ☐ Ecological, evolutionary & environmental sciences

For a reference copy of the document with all sections, see nature.com/documents/nr-reporting-summary-flat.pdf

Life sciences study design

All studies must disclose on these points even when the disclosure is negative.

Sample size	Data was analyzed from 3 replicates (sample size) based on standard sample size used for statistical calculation in biological experiments
Data exclusions	No data were excluded
Replication	All experiments were repeated at least 3 times. Average was reported in the manuscript. Standard deviation of the data was calculated and reported in the form of error bars.
Randomization	Worms from each condition that were imaged under microscope were randomly selected from agar plates.
Blinding	No blinding was used in these experiments. The experimental protocol was identical for worms exposed to different inducer combinations, and data analysis is automated and requires no input from investigators.

Reporting for specific materials, systems and methods

We require information from authors about some types of materials, experimental systems and methods used in many studies. Here, indicate whether each material, system or method listed is relevant to your study. If you are not sure if a list item applies to your research, read the appropriate section before selecting a response.

Materials & experimental systems

n/a	Involved in the study	
☒	☐	Antibodies
☒	☐	Eukaryotic cell lines
☒	☐	Palaeontology and archaeology
☐	☒	Animals and other organisms
☒	☐	Human research participants
☒	☐	Clinical data
☒	☐	Dual use research of concern

Methods

n/a	Involved in the study	
☒	☐	ChIP-seq
☒	☐	Flow cytometry
☒	☐	MRI-based neuroimaging

Animals and other organisms

Policy information about studies involving animals; ARRIVE guidelines recommended for reporting animal research

Laboratory animals	Caenorhabditis elegans hermaphrodites age between L1 larval stage and day-1 adult stage (strain N2, strain SD10184); Escherichia coli (strain BL21, strain BL21(DE3))
Wild animals	No wild animals were used in the study
Field-collected samples	No field collected samples were used in the study
Ethics oversight	No ethical approval or guidance was required since no vertebrate animal was used in the study

Note that full information on the approval of the study protocol must also be provided in the manuscript.
